# Mesoporous Silica-Loaded PCL-CHT Hybrid Membranes for Skin Regeneration

**DOI:** 10.1021/acsami.5c09164

**Published:** 2025-08-11

**Authors:** Simona Salerno, Sabrina Morelli, Andrea Vardè, Marzia De Santo, Camilla Longobucco, Angelica Spadafora, Gianluca Dell’olio, Francesca Giordano, Catia Morelli, Antonella Leggio, Luigi Pasqua, Loredana De Bartolo

**Affiliations:** † 9327 Institute on Membrane Technology, National Research Council of Italy, ITM-CNR, via P. Bucci, cubo 17/C, Rende, CS I-87036, Italy; ‡ Department of Pharmacy, 18950 Health and Nutritional Sciences, University of Calabria, via P. Bucci, Rende, CS 87036, Italy; § NanoSiliCal Devices srl, University of Calabria, Rende, CS 87036, Italy; ∥ Department of Environmental Engineering, University of Calabria, via P. Bucci, Rende, CS 87036, Italy

**Keywords:** hybrid membranes, mesoporous silica nanoparticles, chitosan, polycaprolactone, human keratinocytes

## Abstract

An innovative multifunctional membrane, combining polymeric materials with inorganic nanoparticles and bioactive molecules, was developed for skin tissue application. The strategy was to synthesize a hybrid polymeric/silica membrane in which SiO_2_ nanoparticles are dispersed inside the membrane matrix. To this end, hexagonal calcined mesoporous silica nanoparticles (MSNs) with a uniform structure, 187.6 ± 4.6 nm diameter, and 5.1 nm pore size were synthesized to accommodate molecules of pharmaceutical interest in the silica mesopores. MSNs were then loaded with daidzein, a prominent isoflavone well-known for its anti-inflammatory, antioxidant, and antidiabetic activity, through chemical-physical interactions to investigate its role as a drug carrier. The hybrid membranes were created by combining chitosan (CHT) and polycaprolactone (PCL) polymers with mesoporous silica nanoparticles, optimizing the polymer-to-silica molar ratio up to 5:1, for which enhanced hydrophilicity (WCA = 55.5 ± 2.9°), moisture permeability (WVTR = 32.2 ± 4.4 g/m^2^·h), and swelling capacity (68 ± 11%) were achieved. Drug release studies on the hybrid membrane incorporating daidzein-preloaded silica confirmed sustained delivery of the active compound, releasing 88.9 ± 0.9 μM/cm^2^ after 48 hours. The physical-chemical and morphological-structural properties of the membranes favored the adhesion and growth of human keratinocytes, providing biomimetic cues to facilitate epidermal maturation. In the developed epidermal models, oxygen consumption, which is representative of an active cellular metabolic state, rises over time, leveling off at day 7. The highest oxygen uptake activity was observed in the hybrid membrane PCL-CHT/MSN, achieving values of 161 ± 3 μmol/L at day 11. Hybrid epidermal-membrane constructs enhance keratinocyte proliferation and differentiation, as evidenced by specific cytokeratins, matrix metalloproteinases, and cyclin D1 expression, suggesting improved stratification and epidermal remodeling.

## Graphical abstract

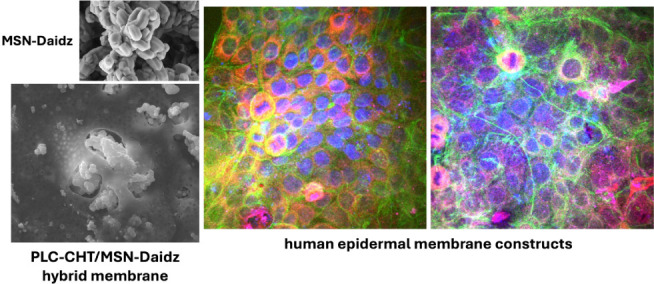

## Introduction

Skin tissue engineering requires the development of multifunctional materials capable of replacing damaged or diseased skin while actively supporting the regenerative process. An ideal biomaterial should facilitate cell migration, proliferation, and differentiation, enable the controlled delivery of bioactive molecules, and provide a protective barrier to prevent transepidermal water loss and microbial infiltration. Natural polymers such as chitosan,^1^ hyaluronic acid,^2^ silk fibroin,^3^ collagen,^4^ and gelatin^5^ have been widely reported to exhibit superior biocompatibility, making them particularly suitable for applications in skin tissue engineering. Chitosan (CHT) stands out among these materials for its great potential in wound dressing due to its biocompatibility, biodegradability, and nontoxic properties. Given its ability to be shaped into various forms, hydrophilic character, pH-dependent cationic nature, and its tendency to interact with anionic glycosaminoglycans (GAGs), heparin, proteoglycans, and nucleotides, CHT is particularly suitable as a biomaterial for skin repair and regeneration.^6,7^ Many studies have reported the benefits of chitosan and its derivatives for skin wound healing, including desirable pharmacological actions (e.g., antibacterial, anti-inflammatory, hemostatic, and skin regenerative properties);^8,9^ effective water absorption and retention capabilities; and the presence of amino (−NH_2_) and hydroxyl (−OH) groups on its molecular chains, which enable the grafting of additional groups and chemical components to enhance specific biological functions.^10^ CHT has been reported to accelerate wound healing by enhancing the recruitment of inflammatory cells and fibroblasts via *N*-acetyl-β-d-glucosamine, which is released during the gradual degradation of CHT.^10^ Although commercial CHT wound dressings have been developed and used as wound-dressing materials, concerns have consistently been raised about their inadequate structural integrity, attributed to poor mechanical properties as well as limited thermal and chemical stability. On the other hand, synthetic polymers (e.g., poly­(vinyl alcohol) (PVA), poly­(l-lactide) (PLLA), polycaprolactone (PCL), and poly­(l-lactide-*co*-caprolactone) (PLCL)^11–13^ have been explored for their elasticity, biodegradability, and mechanical strength. PCL is an FDA-approved resorbable aliphatic polyester that has been widely used as a matrix material, although its application is frequently restricted owing to limited bioactivity, slow degradation rate, and acidic degradation products. An interesting approach to develop a biomaterial with the desired properties overcoming the bottlenecks associated with the individual polymers is to integrate the physical-chemical properties and processability of a synthetic polymer such as PCL with the biocompatibility and bioactive properties of CHT by blending synthetic polymers with natural polymers. PCL-CHT blends have been tested for skin applications as a potential wound dressing. Previously, we synthesized biodegradable membranes of chitosan, polycaprolactone, and a polymeric blend of PCL and CHT for the development of in vitro dermal–epidermal membrane systems by coculturing human keratinocytes with skin-derived stem cells.^14,15^ PCL-CHT membranes promoted the creation of specific epidermal strata or a fully proliferative epidermal multilayer system. Moreover, these membranes promoted the epidermal and dermal differentiation of skin stem cells with high expression of specific cytokeratins and the deposition of the ECM protein fibronectin, respectively.

To improve skin regeneration, various inorganic particles, including silica nanoparticles, attract great interest for their bioactive reaction with tissue.^16^ It has been shown that silicate micro/nanoparticles in contact with a cellular system enhance biological activities such as immunoregulation and angiogenesis, which are triggered by bioactive ions.^17^ Silica particles are known to accelerate the proliferation of fibroblasts, processing temperature, and biodegradability. For wound healing, mesoporous silica nanoparticles decorated with ceria nanocrystals were used as a reactive oxygen species (ROS)-scavenging tissue adhesive, leading to reduced inflammatory responses, limited scar formation, and rapid wound closure.^18^ These effects were attributed to the decreased ROS levels and the nanobridging effect between the nanoparticles and the wound bed.

Silica nanoparticles are also known to be good drug carriers because of their high drug-loading efficiency, which is attributed to their high specific surface area, mesoporous structure, organized porosity, large pore volume, and stable dispersion in aqueous media, making them suitable for incorporating therapeutic molecules.^19^ Additionally, the surface of mesoporous nanoparticles offers several opportunities for functional group grafting and the attachment of various therapeutic macromolecules, enhancing their versatility in the biomedical field.^20,21^ Mesoporous silica represents a foundational nanoarchitecture that enables precise engineering in the design and development of advanced functional nanostructured devices for nanomedicine applications.^22,23^

Inspired by the successful use of PCL-CHT membranes in epidermal–dermal systems and the potential bioactive role of silica nanoparticles, we developed an innovative multifunctional membrane combining polymeric materials with inorganic nanoparticles and bioactive molecules that, from a tissue engineering perspective, is urgently required in the treatment of cutaneous damage. The strategy was to develop hybrid PCL-CHT silica membranes in which SiO_2_ nanoparticles are dispersed inside the membrane matrix. To this end, hexagonal mesoporous silica SBA-15 with a uniform structure, narrow pore size distribution, and large surface area was synthesized to accommodate molecules of pharmaceutical interest in the silica mesopores. Calcined SBA-15 mesoporous silica nanoparticles (hereafter referred to as MSNs) were loaded with daidzein through chemical-physical interactions to investigate their role as drug carriers. Daidzein (4’,7-dihydroxylisoflavone) (Daidz) is a prominent isoflavone of the flavonoid subclass that is widely distributed in Leguminosae plants and various medicinal herbs. It is well-known for its anti-inflammatory, antioxidant, antidiabetic, and cardiovascular activity. Moreover, it shows the ability to modulate several physiological processes, such as cardiovascular function, bone metabolism, and inflammatory disease, supporting human health wellness.^24–26^

The ability of the developed SiO_2_-based hybrid membranes (PCL-CHT/MSN) to promote cell adhesion, differentiation, and functions was evaluated in vitro using human keratinocytes, which potentially can retain the ability to form multilayered, stratified epidermal sheets.

## Materials and Methods

### Materials

Tetraethyl orthosilicate (TEOS), Pluronic P123, ethanol, and dimethyl sulfoxide (DMSO) were obtained from Merck/Sigma-Aldrich (Milan, Italy). Poly­(ε-caprolactone) (PCL, Mn 70–90 kDa, determined by GPC), chitosan (CHT; MW ∼150 kDa, degree of deacetylation 90–95%), lipase from , human lysozyme, and phosphate-buffered saline (PBS) were all obtained from Sigma-Aldrich (Milan, Italy).

The human keratinocyte cell line HaCaT was provided by CLS – Cell Lines Service (Eppelheim, Germany). Phalloidin conjugated to Alexa Fluor 488 and DAPI were supplied by Molecular Probes, Inc. (Waltham, MA, USA). A goat polyclonal antibody targeting human cytokeratin 1 (CK1), a mouse monoclonal antibody against cytokeratin 18 (CK18), Daidzein, and β-actin antibody (clone sc-69879) were obtained from Santa Cruz Biotechnology, Inc. (Heidelberg, Germany). Secondary antibodies, including Cy3-conjugated AffiniPure donkey antimouse IgG and Cy5-conjugated AffiniPure donkey antigoat IgG, were purchased from Jackson ImmunoResearch Europe Ltd. (Cambridge, UK).

Primary antibodies specific for cyclin D1 (clone E3P5S), integrin β1 (clone D6S1W), matrix metalloproteinases MMP1 (clone EAS9N), and MMP2 (clone D4M2N) were obtained from Cell Signaling Technology, Inc. (Leiden, The Netherlands). The antibody against matrix metalloproteinase MMP9 (clone JA80-73) was purchased from Invitrogen (Waltham, MA, USA). IRDye secondary antibodies, the Odyssey FC Imaging System, and the Image Studio Lite v5.2 software were all obtained from LI-COR Biosciences GmbH (Bad Homburg, Germany).

### Synthesis of MSNs

MSN powder was prepared using poly­(ethylene oxide)-poly­(propylene oxide)-poly­(ethylene oxide) triblock copolymer Pluronic surfactant P123 as the structure directing agent and tethaethylorthosilicate (TEOS) as the silica source (Figure S1). For preparing MSN, Pluronic (16 g) was dissolved in a mixture of ultrapure water and 37% HCl (480 mL of 2 M HCl) under magnetic stirring at room temperature until complete dissolution of the polymer. Then, TEOS (36.4 mL) was added dropwise to the acidic surfactant solution with continuous stirring. The resulting mixture was stirred at 40 °C for 20 h in a sealed beaker. The molar ratio employed in the synthesis was TEOS:P123:H_2_O:HCl = 1:0.017:163:5.9. Subsequently, stirring was stopped, and the reaction mixture was transferred to an oven and aged under static conditions at 80 °C for 48 h. The resulting solid was recovered by vacuum filtration, thoroughly washed with ethanol, and finally dried in an oven at 70 °C for 24 h, yielding a white powder. The dried material was then calcined in a muffle furnace by heating at a rate of 5 °C/min up to 450 °C and maintaining this temperature for 4 h to remove the organic template. A final yield of 9.33 g of calcined MSN powder was ultimately recovered.

### Daidzein Loading on MSN

Daidzein was loaded onto the mesoporous silica nanoparticles using a two-step impregnation procedure (Figure S1). This approach was chosen to enhance the loading efficiency of daidzein by maximizing its interaction with the mesoporous network.

In the first step, 375 mg of Daidz was dissolved in a few drops of DMSO to ensure complete solubilization, followed by the addition of ethanol (75 mL) under magnetic stirring. The resulting drug solution was stirred at room temperature for 2 h. Subsequently, calcined MSN powder (1.5 g) was added to the solution, and the resulting suspension was stirred at room temperature for 24 h. The mixture was then filtered under vacuum, and the filtrate was collected for the second loading step.

In the second step, an additional amount of 375 mg of Daidz was added to the recovered filtrate. After 1 h of stirring, the previously collected MSN-Daidz nanoparticles were introduced into the drug solution. The suspension was stirred for an additional 72 h at room temperature. After this period, stirring was stopped, and the suspension was filtered. The collected nanoparticles were then dried in an oven at 45 °C for 24 h, yielding 1.93 g of the MSN-Daidz sample.

All reagents were commercially available in analytical grade and were used without further purification. Solvents were purified following established laboratory procedures and freshly distilled before use.

### MSN and MSN-Daidzein Characterization

Thermogravimetric analysis (TGA) was carried out using a Jupiter STA 449 F5 instrument (NETZSCH-Gerätebau GmbH, Germany) in the range of 20–850 °C at a ramp rate of 10 °C/min in air with a flow rate of 10 mL/min. Ultrapure water was obtained by using the Milli-Q water system from Millipore (Burlington, MA, USA). Fourier transform infrared (FTIR) spectra were recorded by using an FT/IR-4600 FT-IR spectrometer (Jasco, Germany). The samples were mixed with KBr, and the IR spectra were obtained between 500 and 4000 cm^–1^. The ordered mesoporous framework of the synthesized materials was examined by small-angle powder X-ray diffraction (XRD) on a MiniFlex 600 diffractometer (Rigaku Holding Corporation, Tokyo, Japan), operating at 40 kV and 15 mA, employing Ni-filtered Cu Kα radiation (λ = 1.54059 Å) in the 2θ range of 0.4–7° with a scan speed of 0.3 deg/min. Scanning electron microscopy (SEM) was performed on an ESEM FEG QUANTA 200 scanning electron microscope. The samples were prepared by placing MSN powder on double-sided carbon adhesive tape mounted on the sample holder. Transmission electron microscopy (TEM) was carried out with a Jeol 1400 Plus electron microscope, operating at an acceleration voltage of 80 kV. The size distribution of MSNs was determined using dynamic light scattering (DLS) analysis with a 90 Plus Particle Size Analyzer (Brookhaven Instruments Corporation, New York, USA). A 0.1 % wt suspension of MSNs was prepared in distilled water and sonicated in an ultrasonic bath for 5 min. DLS measurements were performed at 25 °C.

Porosity and specific surface area were estimated by N_2_ adsorption–desorption at −196.15 °C and were collected using a Tristar II Plus 3.02 porosimeter (Micromeritics Instruments Corporation, Norcross, GA, USA) under continuous adsorption conditions. Samples were pretreated at 120 °C in the degassing system for 150 min before the analysis. The BET specific surface area was calculated based on the adsorption data in the relative pressure range of 0.05–0.30. The pore volume was evaluated at a relative pressure of 0.96. The pore size distribution was determined from the desorption branch of the N_2_ adsorption isotherms using the Barret–Joyner–Halenda (BJH) model. Zeta potential analysis was performed using Zetasizer Ultra (Malvern-Panalytical, Worcestershire, UK). Measurements were conducted in ultrapure water, with a viscosity of 0.8872 cP and a refractive index of 1.330 at 25 °C. The thermostatting time was 120 s, the dielectric constant of the dispersing medium was 78.5, and three replicate measurements were taken for each sample. Data are expressed as the arithmetical mean ± standard deviation.

### Membrane Preparation

PCL-CHT and hybrid PCL-CHT/MSN membranes were prepared by a liquid-induced modified phase inversion technique (Figure S1). PCL and CHT, in a ratio of 90/10 (14 wt %), were dissolved in a formic acid (FA)/acetic acid (AA) 6:4 (w/w %) mixture until complete dissolution. For the hybrid membranes, silica nanoparticles MSN (or MSN-Daidz) were added to the polymeric PCL-CHT solution, with the molar ratio of polymer/silica varied at 20:1, 15:1, 10:1, 7.5:1, and 5:1. The hybrid PCL-CHT silica solution was stirred continuously for an additional 6 h and subsequently sonicated for 30 min to ensure complete homogenization and nanoparticles dispersion. Both the polymeric and hybrid solutions were then cast onto glass plates by using a casting knife, with a controlled thickness of 250 μm. The cast films were allowed to evaporate at room temperature for 2 min to initiate partial solvent removal. Following this, the glass plates were gradually immersed in a nonsolvent bath consisting of NaOH solution at room temperature. This immersion induced phase inversion via solvent–nonsolvent exchange, leading to the formation of a membrane sheet through demixing of the polymer solution. The NaOH solution functions both as a nonsolvent to initiate phase inversion and as a chemical agent to deprotonate amino groups of chitosan and to induce its precipitation, which is essential for achieving structural integrity and phase compatibility within the PCL–CHT and PCL-CHT/MSN membranes. The immersion of the cast film into the NaOH coagulation bath facilitates a rapid solvent–nonsolvent exchange between the acidic casting solution and the alkaline aqueous medium. This exchange drives phase separation, leading to the formation of a polymer-rich phase, which constitutes the solid membrane matrix, and a polymer-lean phase, which contributes to pore formation. The kinetics of this exchange affect membrane porosity, morphology, and mechanical stability.

Following the completion of the phase separation process, the membranes were carefully detached from the glass substrates. All membranes underwent multiple rinsing steps with distilled water to eliminate residual chemicals, followed by a final drying process prior to further analysis.

### Membrane Characterization

Membranes were characterized to evaluate their structural and physical-chemical properties. The surface and cross-sectional morphology of the investigated membranes were observed by a High-Resolution Scanning Electron Microscope (HRSEM, model CrossBeam 350 ZEISS – Germany) at 5 kV of energy, following prior coating with graphite. Energy-dispersive spectroscopy (EDS) analysis was also performed on the same membrane samples to assess the silica weight and atomic percentage in several membranes.

The membrane thickness was measured using a digital micrometer (Carl Mahr 40E, Germany) by averaging ten measurements taken in different areas.

The wettability of the membranes was determined by water contact angle (WCA) measurements using a contact angle meter (KSV Instruments, Ltd., Helsinki, Finland) and the sessile drop method. The WCA values are the mean of 30 measurements.^27^

The surface zeta potential of PCL-CHT and PCL-CHT/MSN membranes was assessed through electrokinetic analyses carried out by a SurpassTM 3 (Anton Paar) analyzer, employing the streaming potential and streaming current methods with a 5 mmol/L KCl aqueous solution in the pH range of 5.5–8.5.

The daidzein loading in the hybrid PCL-CHT/MSN-Daidz membranes, expressed as the weight of Daidz loaded per surface area of the membrane (μg/cm^2^_membr_), was evaluated by dissolving 4 cm^2^ membrane samples in a solvent mixture of chloroform/dimethylformamide (4:1) and analyzing them using UV–vis spectrophotometry. Daidzein release was assessed by incubating 4 cm^2^ of membrane samples, after UV sterilization, in 2 mL of phosphate-buffered saline (PBS)/ethanol (EtOH) (3:1) solution at pH 7.4 and 37 °C, with the incubation solution being changed and analyzed at specific time intervals using UV–vis spectrophotometry at a wavelength of 252 nm. To assess the daidzein release mechanisms and kinetics, the Higuchi model and Korsmeyer–Peppas model were used to fit the in vitro release data up to 24 h, by using the following equations:

1 $$\mathrm{Higuchi model}\;M_{t}/M_{\infty }=K_{H}t^{1/2}$$

2 $$\mathrm{Korsmeyer-Peppas model}\;M_{t}/M_{\infty }=K_{KP}t^{n}$$

where *M*_t_ */M*_*∞*_ is the fraction of drug released at each time point (*t*), *M*_t_ is the amount of drug released at time *t*, *M*_*∞*_ is the amount of drug released after time ∞, *K*_H_ and *K*_KP_ represent the Higuchi and Korsmeyer–Peppas release kinetic constants, respectively, and *n* is the diffusional exponent. From the linear regression of these plots, correlation coefficients and kinetic constants (*R* ^2^, *K*_H_, *K*_KP_, and *n*) were calculated and compared.

The membrane degradation was evaluated by using PBS at pH 7.4 and 37 °C, in the presence of 0.1% NaN_3_ to simulate the physiological environment, and in an enzymatic solution to mimic the natural biodegradation process that breaks down polymer chains into smaller molecules. Lipase from (0.12 U/mL) and human lysozyme (1300 U/mL) were used, respectively, for the enzymatic solution. Six membrane samples (4 cm^2^) from each batch were dried in a vacuum oven at 40 °C for 5 h, and then, their initial weight (*W*_i_) was precisely measured. Thereafter, the samples were immersed in 1 mL of enzymatic or physiological solution at pH 7.4 and 37 °C. At regular time intervals, the samples were withdrawn from the degradation medium. Then, they were washed with distilled water and dried in a vacuum oven at 40 °C for 5 h, and their final weight (*W*_f_) was precisely measured. After weighing, the enzymatic solution was refreshed, and the membranes were immersed again until the next weight measurement. The weight loss index (*W*_loss_ %) was determined by the following equation:

3 $$W_{loss}\%=\frac{W_{i}-W_{f}}{W_{i}}\times 100$$

The swelling index of the membranes was evaluated in PBS by using a gravimetric method. Eight membrane samples (1.5 cm^2^) from each batch were dried in a vacuum oven at 40 °C for 5 h, and then, their initial weight (*W*_d_) was precisely measured. The samples were then immersed in 2.5 mL of PBS containing 0.1% NaN_3_ at pH 7.4 and 37 °C. At predetermined time intervals, the membranes were carefully removed from the medium, gently blotted with filter paper to eliminate excess surface moisture, and immediately weighed to obtain the swollen weight (*W*_w_). The swelling ratio was calculated according to the following equation:

4 $$Swelling\;Index\left(\%\right)=\frac{W_{w}-W_{d}}{W_{d}}\times 100$$

For the water vapor transmission rate (WVTR) measurements, six circular membrane samples (2.5 × 10^–4^ m^2^) from each batch were mounted onto the opening of cylindrical cups filled with 2 mL of distilled water. The interface between the sample and the cup was hermetically sealed to prevent vapor leakage. The assembled system was placed in an incubator maintained at 37 °C and 85–90% relative humidity. The WVTR was calculated by monitoring the variation in water mass (Δ*w*) with measurements taken at regular time intervals (Δ*t*) and expressed as grams of water vapor per square meter (*A*) per hour, according to the following equation:

5 $$WVTR=\frac{\Delta w}{A\times \Delta t}$$

### Cell Cultures

Human keratinocytes (HaCaT) with a 38 population-doubling level were seeded at a cell density of 6.5 × 10^5^ cell/cm^2^ on membranes that had been previously UV-sterilized. The cells were maintained in the DMEM medium containing 4500 mg/L glucose and GlutaMAX Supplement (Sigma-Aldrich, Milan, Italy), enriched with 10% FCS, 50 g/mL streptomycin, and 100 U/mL penicillin (Life Technologies, Carlsbad, CA). The cells were incubated at 37 °C in a 5% CO_2_/20% O_2_ atmosphere (v/v) with 95% relative humidity and maintained for up to 14 days, with the culture medium being changed every 48 h.

### Cell Morphology

Cell morphology was examined by confocal laser scanning microscopy (CLSM, Fluoview FV300, Olympus Italia) with appropriate immunostaining after 7 days of culture on the different membranes. After three washes with PBS, cellular samples were fixed in paraformaldehyde, permeabilized with Triton X-100, and saturated with serum as previously described.^28^ The cytoskeletal protein actin was stained with Alexa 488-conjugated phalloidin incubated for 30 min. A goat polyclonal antibody raised against human CK1 and Cy5-conjugated AffiniPure donkey antigoat IgG was used to visualize CK1, while a mouse monoclonal antibody raised against human CK18 and Cy3-conjugated AffiniPure donkey antimouse IgG was used to visualize CK18.

Primary and secondary antibodies were incubated at room temperature for 2 and 1.5 h, respectively. Nuclei were counterstained for 30 min with 0.2 g/mL of DAPI.

### Cell Viability and Metabolic Activity

Cell viability and proliferation were assessed by the 3-(4,5-dimethylthiazol-2-yl)-2,5-diphenyl tetrazolium bromide (MTT) test. After 3, 7, and 14 days of culture on the different membranes and treatments, human keratinocytes were incubated in 5 mg/mL of MTT solution for 4 h at 37 °C. The yellow tetrazolium MTT salt was reduced by mitochondrial dehydrogenase in living cells to purple formazan crystals, which were extracted by dissolving the cells with 1 mL per sample of a lysis solution containing 10% sodium dodecyl sulfate and 0.6% acetic acid in DMSO, under mild stirring for 30 min at 37 °C. The formazan product was then quantified by spectrophotometry at a wavelength of 570 nm. The cell metabolic activity was evaluated by investigating the oxygen consumption of keratinocytes during the culture period. O_2_ concentration in the culture medium was noninvasively detected by using a Sensor Dish Reader (SDR), OxoDish-DW (PreSens Precision Sensing GmbH), which allows real-time monitoring of dissolved oxygen with a resolution of ±0.4% O_2_ at 20.9% O_2_ and a response time <30 s.

### Cell Cycle Analysis

To analyze cell cycle distribution, cells cultured for up to 7 days on the developed membranes were harvested, pelleted, washed once with PBS, and fixed in 50% methanol overnight at −20 °C. Cells were then stained with a solution containing 50 μg/mL propidium iodide (PI) in PBS, 20 U/mL RNase-A, and 0.1% Triton. Cell phases were estimated as a percentage of a total of 10000 events. Samples were analyzed with CytoFLEX flow cytometry (Beckman-Coulter, Milan, Italy).

### Western Blotting (WB)

Cells cultured for up to 7 days on the developed membranes were trypsinized, harvested, and lysed to obtain total protein extracts by lysing the cells in RIPA buffer supplemented with a mixture of protease inhibitors (aprotinin, phenylmethylsulfonyl fluoride, and sodium orthovanadate). After separation on an SDS-PAGE gel and transfer to nitrocellulose membranes, proteins were detected using specific antibodies: cyclin D1, integrin β1, and matrix metalloproteinases MMP1, MMP2, MMP9, and β-actin.

### Gelatin Zymography Assay

Gelatinolytic activity of MMP2 and MMP9 and their quantities in conditioned media were analyzed by gelatin zymography. Samples of medium collected after 7 days of cell culture on the developed membranes and under different treatments were centrifuged to remove cellular debris, diluted 1:20 in culture media, and precipitated in acetone. Fifteen microliters of each sample was then separated on SDS–polyacrylamide gel electrophoresis containing 0.1% gelatin. The gel was washed with washing buffer (50 mM Trizma base pH 7.5, 10 mM CaCl_2_, 2.5% Triton X-100), incubated overnight in incubation solution (50 mM Trizma base pH 7.5, 10 mM CaCl_2_, 1% Triton X-100) at 37 °C, and stained with Coomassie blue. The gelatinolytic activity of MMP2 and MMP9 was evaluated by the presence of areas of degradation visible as clear bands on the dark gel.

### Statistical Analysis

Statistical analysis was performed using ANOVA followed by the Bonferroni *t* test (statistical significance: *p* < 0.05).

## Results and Discussion

### MSN Properties

The XRD powder pattern of the MSN sample is presented in Figure 1. One prominent peak was observed at 2θ = 0.62°, followed by a weak peak at 2θ = 0.88°, which is typical of the hexagonal array parallel pore structure of the nanomaterial.^29^

**Figure 1:**
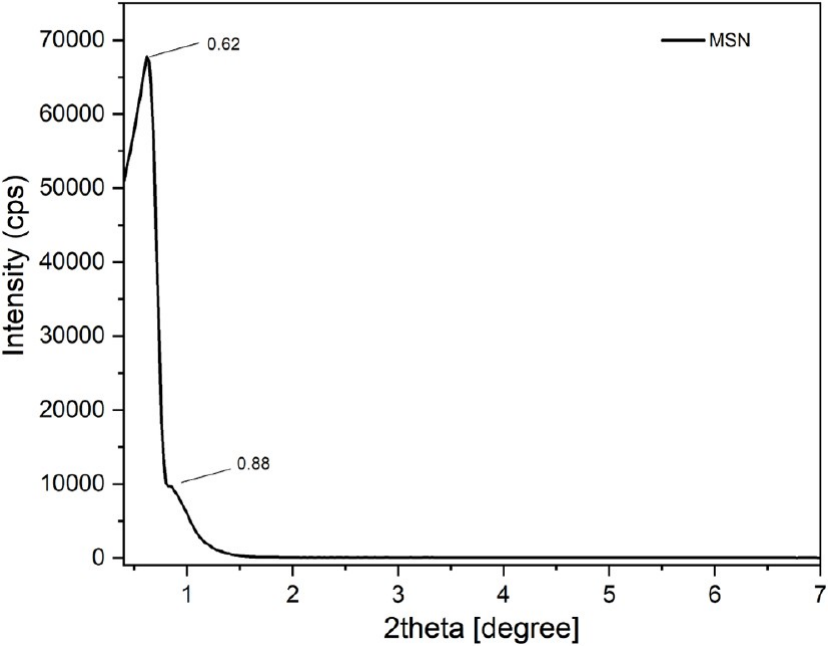
XRD pattern of the MSN sample.

Morphological properties of nanoparticles were determined by using SEM and TEM imaging. We measured nanoparticles with dimensions of approximately 145 × 230 nm, which are representative of the average size observed in the SEM image shown in Figure 2. The SEM image clearly revealed well-defined nanoparticles with a cylindrical shape (Figure 2a).

**Figure 2:**
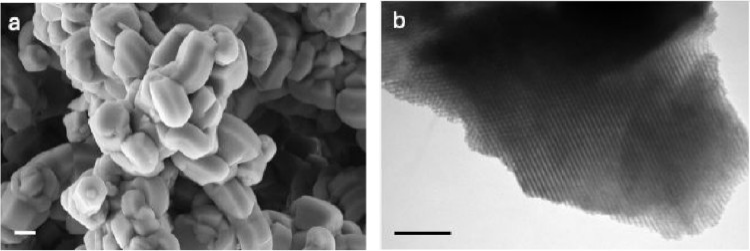
Morphological properties of nanoparticles: SEM (a) and TEM (b) micrographs of the MSN sample. Scale bar: 1 μm (a) and 0.1 μm (b).

The TEM image in Figure 2b confirmed the ordered structure of the material, revealing a periodically well-organized hexagonal arrangement of mesopores, characteristic of the SBA-15 family.^30^ The distinct, uniform, and parallel stripes are indicative of the highly ordered mesoporous structure of the synthesized material, suggesting that the mesoporous framework retains its structural integrity after calcination.^31^

The particle size distribution is reported in Figure 3. The average hydrodynamic diameter, determined by DLS measurements, is 187.6 ± 4.6 nm, with a polydispersity index of 0.30. These DLS results are consistent with the mean particle size observed in the SEM analysis.

**Figure 3:**
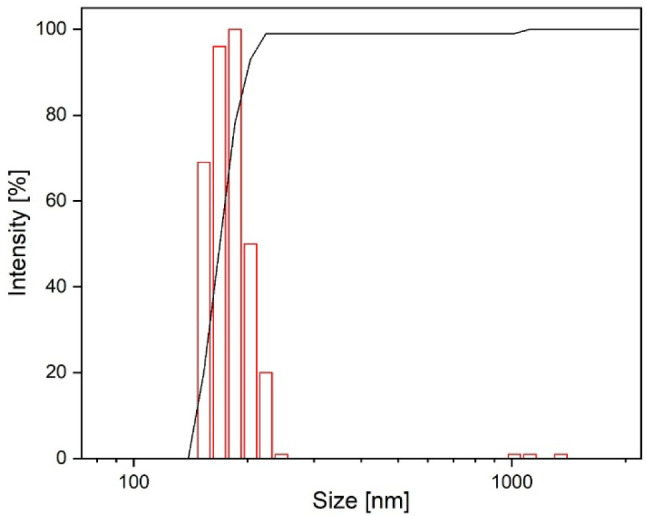
Particle size distribution of the MSN sample.

Considering its well-documented anti-inflammatory and antioxidant properties, daidzein was employed as a bioactive agent to realize a specific skin layer using human skin cells and a newly developed biodegradable hybrid membrane incorporating mesoporous silica nanoparticles.

Daidzein was loaded onto MSN following the previously described impregnation steps. The drug loading quantification was obtained with thermogravimetric measurements: at high temperatures, the organic drug molecule Daidz decomposes, and the mass loss is proportional to the total drug content in the MSN samples.^32^ According to TGA analysis, the drug loading of Daidz was found to be 31.9%. To the best of our knowledge, Daidzein has not yet been encapsulated in mesoporous silica nanoparticles. Reported delivery systems for daidzein include lipid nanoparticles^33^ and hydroxyapatite nanoparticles.^34^

N_2_ adsorption–desorption isotherms for MSN and daidzein-loaded nanoparticles (MSN-Daidz) are presented in Figure 4. All the isotherms exhibit a type-IV pattern with a well-defined hysteresis loop, characteristic of mesoporous materials with hexagonally ordered pores.^35^ The adsorbed volume increased sharply at relative pressure (*P*/*P* ^0^) ranging from 0.4 to 0.7, indicating a mesoporous structure with a highly homogeneous pore size distribution.

**Figure 4:**
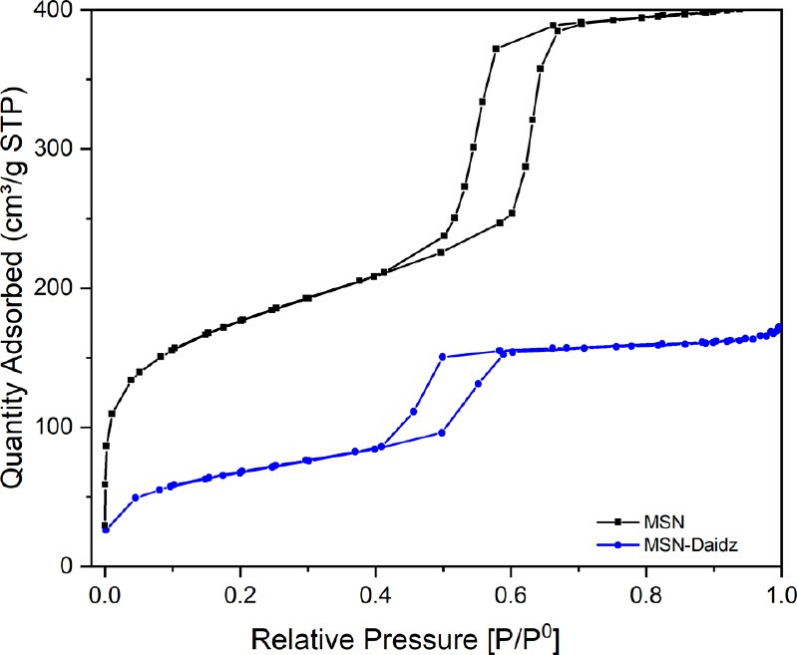
N_2_ adsorption-desorption isotherms of MSN and MSN-Daidz samples.

As displayed in MSN-Daidz isotherms, the inflection point shifted slightly toward lower relative pressures, and the volume of nitrogen adsorbed decreased following drug loading, indicating a reduction in pore size. It is interesting to note that even after the drug loading, the shape of the isotherms was maintained, indicating that the mesoporous ordered structure of samples was preserved. The BET surface area, pore size, and pore volume of MSN are 613 m^2^/g, 5.1 nm, and 0.62 cm^3^/g respectively, as displayed in Table 1. As daidzein was loaded onto MSN, an expected decrease in the surface area, pore volume, and pore size was observed, with reported values of 240 m^2^/g, 0.26 cm^3^/g, and 4.3 nm, respectively (Table 1).

**Table 1:** Detailed Structural Features of the Synthesized Samples

**Sample**	**BET Surface area** [m^2^/g]	**Pore volume** [cm^3^/g]	**Pore size [nm]**
**MSN**	613	0.62	5.1
**MSN-Daidz**	240	0.26	4.3

Pore volume distribution profiles of MSN and Daidz-loaded MSN samples are calculated from the desorption branches of the nitrogen adsorption–desorption isotherms using the Barret–Joyner–Halenda (BJH) method, as shown in Figure 5a. Consistent with TEM observations, both MSN and MSN-Daidz samples exhibited a uniform and narrow pore volume distribution. After drug loading, a slight shift in the pore volume distribution was observed, indicating the incorporation of Daidz within the mesopores of MSN. Pore volume values indicated in Table 1 reflect the pore distributions for both MSN and MSN-Daidz samples.

**Figure 5:**
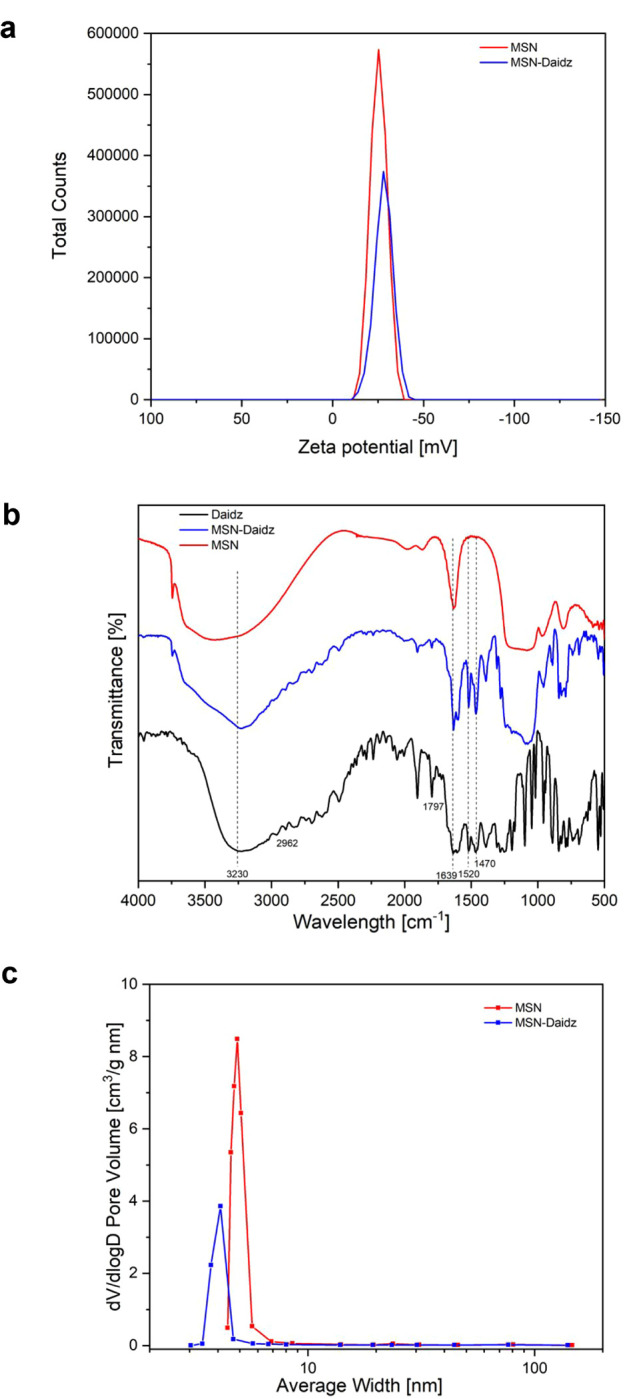
Properties of MSN and MSN-Daidz samples: (a) pore volume distribution, (b) FTIR spectra, and (c) zeta potential pattern.

To confirm the loading of daidzein onto nanoparticles, we studied the FTIR spectra of Daidz, MSN, and MSN-Daidz. The FT-IR spectrum of daidzein, as shown in Figure 5b, reveals a prominent absorption band corresponding to the O–H stretching at approximately 3230 cm^–1^. Additionally, it exhibits absorption bands for C=C stretching vibrations of the benzene ring at 1470 cm^–1^ and 1520 cm^–1^, along with an absorption band for C=O stretching at 1639 cm^–1^.^36^ Compared to MSN, the FT-IR spectrum of MSN-Daidz clearly displays the characteristic absorption peaks of Daidz. A similar absorption pattern can be observed in the range of 2962–1250 cm^–1^. For all silica-based samples, typical Si–O–Si bands in the range 1070–1220 cm^–1^, typical of the silica network, are appreciable. The broad peak at around 3400 cm^–1^ is attributed to the O–H stretching vibration mode.^37,38^

Zeta potential analysis revealed strong electrostatic interactions between nanoparticles and demonstrated good physical stability of the synthesized materials. As a result of drug loading, the zeta potential of MSN shifted from −25.36 ± 0.69 mV to −28.32 ± 0.26 mV, as shown in Figure 5c.

### Hybrid PCL-CHT Silica Membranes

Figure 6 (panel I) shows SEM micrographs of PCL-CHT and hybrid PCL-CHT/MSN membranes from both sides of the membranes. The PCL-CHT and PCL-CHT/MSN membranes exhibited well-interconnected porous structures, which are important for permeability properties. The top and bottom membrane surfaces display noticeably different microstructural features: larger pore sizes are present on the bottom surfaces (facing the glass) compared to the top surfaces. SEM analysis reveals that the hybrid PCL-CHT/MSN membranes possess a heterogeneous morphology, where polycaprolactone and chitosan form the continuous polymer matrix. Within this matrix, mesoporous silica nanoparticles are distinctly distributed, contributing to nano- and microscale porosity. These nanoparticles often appear either partially embedded within the polymer surface or protruding outward, as seen in Figure 6 (panel II). Aggregation of MSNs can lead to the formation of mesopores and micropores, significantly increasing the surface area available for adsorption and facilitating drug loading and controlled release. Additionally, the membranes exhibit a granular or rough-textured surface, attributed to the presence of exposed or partially embedded MSNs, which improves cell attachment. These structural features play a pivotal role in enhancing membrane performance in skin tissue engineering applications. The increase of MSN concentration enhanced the porosity and the presence of micropores and mesopores, achieving optimal dispersion and microporosity in the PCL-CHT/MSN membranes with a molar ratio of 5:1 (Figure S2). The addition of MSNs increased the membrane thickness from 25 ± 1 mm for the PCL-CHT membrane to 66 ± 5 mm for the PCL-CHT/MSN membrane with a molar ratio of 5:1. Moreover, energy-dispersive spectroscopy (EDS) analysis was performed on both surfaces of all investigated membranes to assess their elemental composition and distribution (Figure S3). Table 2 reports the variation in Si atomic and weight percentages in the top and bottom surfaces of the hybrid PCL-CHT/MSN membranes with different molar ratios of polymers:silica. It is possible to observe that the increase of silica concentration gives rise to an increment of the Si atomic and weight percentages, especially at the bottom surface compared to the top one. The highest values of weight (top = 1.6 ± 0.7%, bottom = 2.8 ± 0.7%) and atomic percentages (top = 0.8 ± 0.3%, bottom = 1.4 ± 0.2%) were found on the PCL-CHT/MSN membrane with a molar ratio of 5:1, providing evidence of the substantial amount of silica nanoparticles distributed within the polymer matrix.

**Figure 6:**
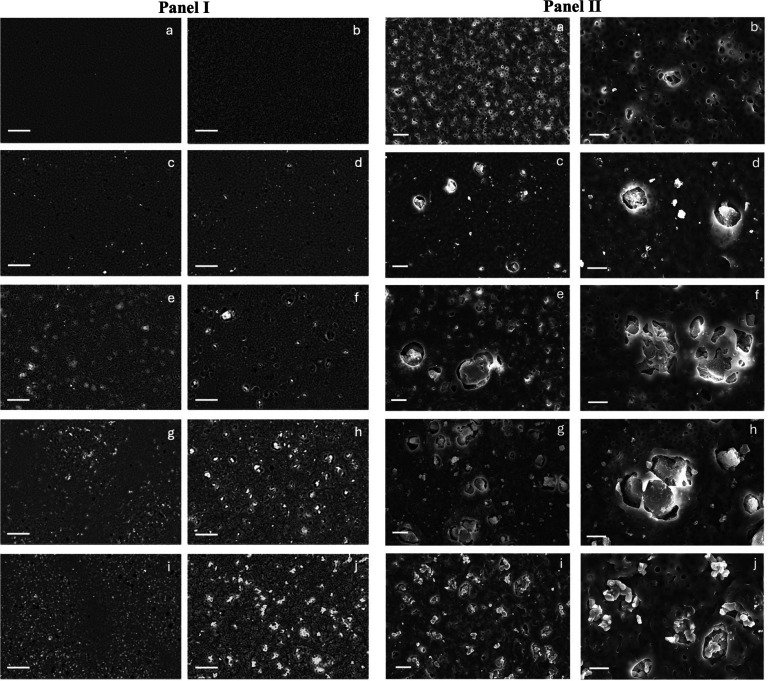
**Panel I**: SEM micrographs in the BSD signal of the top (a, c, e, g, i) and bottom surfaces (b, d, f, h, j) of the PCL-CHT (a,b) and hybrid PCL-CHT/MSN membranes with molar ratios of 20:1 (c,d), 10:1 (e,f), 7.5:1 (g,h), and 5:1 (i,j). Scale bar: 50 μm. **Panel II**: SEM micrographs in the SE signal at different magnifications of the bottom surfaces of the PCL-CHT (a,b) and hybrid PCL-CHT/MSN membranes with molar ratios of 20:1 (c,d), 10:1 (e,f), 7.5:1 (g,h), and 5:1 (i,j). Scale bar: 20 μm (a, c, e, g, i) and 10 μm (b, d, f, h, j).

**Table 2:** Silica Weight and Atomic Percentage of Hybrid PCL-CHT/MSN Membrane Surfaces (Top and Bottom) with Different Molar Ratios of Polymers/Mesoporous Silica Nanoparticles^a^

**PCL-CHT/MSN ratio**	**Si weight % top layer**	**Si weight % bottom layer**	**Si atomic % top layer**	**Si atomic % bottom layer**
20/1	**0.6** ± 0.2	**0.6** ± 0.1	**0.3** ± 0.1	**0.3** ± 0.1
15/1	**0.6** ± 0.2	**0.9** ± 0.2	**0.3** ± 0.1	**0.4** ± 0.1
10/1	**1.0** ± 0.2	**1.5** ± 0.1	**0.4** ± 0.1	**0.7** ± 0.2
7.5/1	**1.1** ±0.4	**1.7** ± 0.6	**0.5** ± 0.1	**0.8** ± 0.2
5/1	**1.6** ± 0.7	**2.8** ± 0.7	**0.8** ± 0.3	**1.4** ± 0.2

a Data are reported as the mean and standard deviation of 8 different EDS analyses per surface on different zones of different samples.

Since surface wettability plays a critical role in cell-biomaterial interactions, we evaluated the water contact angle of the hybrid PCL-CHT/MSN membranes with increasing silica nanoparticle content. The addition of MSNs to the PCL-CHT polymer solution decreased the contact angle from 78.0 ± 2.0° to 71.8 ± 3.2° at the top surface and from 77.3 ± 1.2° to 61.6 ± 3.1° at the bottom surface (Figure 7). The increase of silica nanoparticles added to the dope during membrane preparation reduced the hydrophobicity of the PCL-CHT membrane, achieving a membrane with higher hydrophilic character by using PCL-CHT/MSN with a molar ratio of 5:1 (WCA = 55.5 ± 2.9° at the bottom surface). The WCA difference between the top and bottom surfaces confirmed the asymmetric nature of the membranes, with the bottom surface exhibiting lower contact angle values, consistent with the higher silica content on that side compared to the top surface.

**Figure 7:**
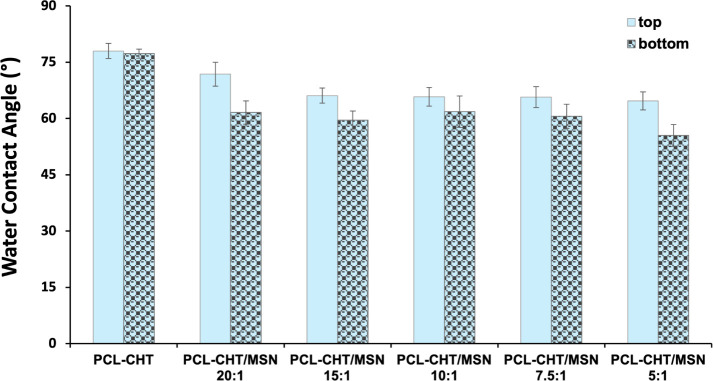
Water contact angles of the top and bottom surfaces of the native membrane without silica (PCL-CHT) and hybrid membranes with mesoporous silica nanoparticles (PCL-CHT/MSN) with different molar ratios of polymers:mesoporous silica nanoparticles.

These findings highlight the suitable chemical-physical and morphological surface properties of the hybrid membranes containing the highest concentration of silica nanoparticles—specifically, the PCL-CHT/MSN membrane with a 5:1 molar ratio. Based on these promising characteristics, we further investigated the surface charge behavior of this membrane in comparison to the native one at different pH levels. Zeta potential measurements were conducted across a range of pH values (5.5 to 8.5), representing physiological and pathological conditions commonly encountered during the wound-healing process. PCL-CHT membranes exhibited a positive zeta potential (+0.7 mV) in an acidic environment (pH 5.5) (Figure S4). The incorporation of MSN increased the zeta potential to +2.7 mV. Both membranes shift toward modestly negative values at pH 6–7 due to chitosan deprotonation and the negative surface charge of silica driving the overall charge. Raising the pH to values of 8.0–8.5, which are normally found in chronic or infected wounds, a further decline to −23 and −24 mV was observed for PCL-CHT and PCL-CHT/MSN membranes, respectively. This dynamic surface behavior makes the membranes particularly attractive for multiphase wound healing and skin tissue engineering applications, supporting cell proliferation and migration while minimizing chronic inflammation. This property enhances long-term tissue integration, allowing for a smart, pH-responsive membrane that adapts to different wound-healing stages, modulates cell-material interactions, and enhances effectiveness in inflammatory environments.

### Daidzein-Preloaded Silica Membranes

MSNs were preloaded with daidzein (MSN-Daidz), a phytoestrogen with antioxidant activity, with the aim of developing a hybrid membrane loaded with an active compound that would be successively released.

During the membrane preparation, a 22 ± 7% loss of daidzein was found in the neutralization and washing solutions collected from six different synthesis batches, thus resulting in a membrane loading efficiency of 78 ± 7%. From the same batches, a daidzein membrane loading of 128 ± 29 μg/cm^2^_membr_ was estimated. The membrane sterilization process in ethanol for 2 h resulted in a significant and almost total loss of the loaded compound (93.1 ± 5.4%), while the process under UV rays for 1 h lost only 6.2 ± 0.2%. The daidzein release was then assessed on membranes previously sterilized under UV rays to determine the effective amount of the active compound available to cells upon contact with the membranes under sterile culture conditions. As shown in Figure 8a the hybrid PCL-CHT/MSN-Daidz membrane released the active loaded compound, reaching a maximum of 45.2 ± 0.9 μg/cm^2^_membr_, equal to 88.9 μM/cm^2^_membr_ after 48 h. The first 24 h of the experimental release data was analyzed according to kinetic mathematical models (Table 3). The Higuchi model showed an excellent fit (*R* ^2^ = 0.966) and a high release constant (*K*_H_ = 19.47), indicating that the release kinetics are primarily governed by a Fickian diffusion-controlled process, consistent with the assumption of the Higuchi model for drug diffusion from a matrix system. The Korsmeyer–Peppas model yielded a slightly lower correlation (*R* ^2^ = 0.912) and release constant (*K*_KP_ = 12.03). These findings are consistent with previous studies involving polymeric membranes functionalized with azithromycin-loaded SBA-15, where similar release parameters and mechanisms were reported.^39^ Importantly, the diffusional exponent *n* = 0.72 of the Korsmeyer–Peppas model suggests a transport mechanism governed by a combination of Fickian diffusion coupled with polymer matrix swelling or relaxation. This suggests that while diffusion remains the dominant mechanism, structural modifications of the polymeric membrane matrix also influence the release process. Such behavior is typical in complex delivery systems where both the mesoporous structure and the physical-chemical interactions between the polymer matrix and the active compound influence the release kinetics, thereby highlighting the synergistic role of the MSNs and the polymer matrix of the hybrid PCL-CHT/MSN-Daidz membrane in providing controlled and sustained drug release, particularly for hydrophobic or low-bioavailability compounds like daidzein.

**Table 3:** Release Modeling Parameters

Models	*R* ^2^	*K*	*n*
**Higuchi**	0.966	*K*_H_ 19.47	
**Korsmeyer**–**Peppas**	0.912	*K*_KP_ 12.03	0.72

**Figure 8:**
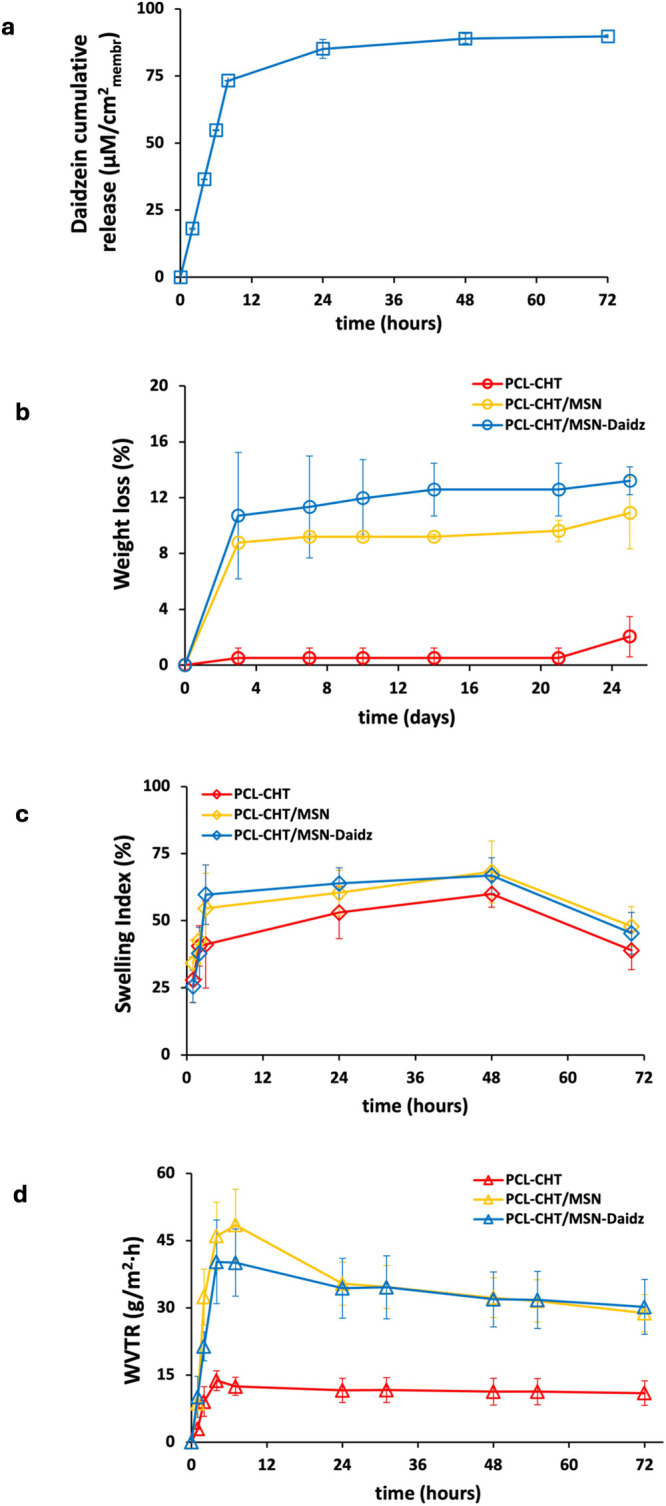
(a) Daidzein cumulative release per membrane area unit (μM/cm^2^_membr_) as a function of time by hybrid membranes with daidzein-preloaded mesoporous silica nanoparticles (PCL-CHT/MSN-Daidz). (b) Weight loss, (c) swelling index, and (d) water vapor transmission rate of native membranes without silica (PCL-CHT), hybrid membranes with mesoporous silica nanoparticles (PCL-CHT/MSN), and hybrid membranes with daidzein-preloaded mesoporous silica nanoparticles (PCL-CHT/MSN-Daidz) at different time intervals in phosphate-buffered saline at pH 7.4 and 37 °C.

The biodegradability of membranes was then investigated to evaluate the percentage of weight loss over time for the native membranes without silica (PCL-CHT), hybrid membranes with silica (PCL-CHT/MSN), and hybrid membranes with daidzein-preloaded silica (PCL-CHT/MSN-Daidz) (Figure 8b). In the enzymatic solution containing lipase and lysozyme, all of the membranes are almost completely degraded within the first 48 h. In contrast, without the enzymes, in a buffer simulating physiological fluids, the native PCL-CHT membrane shows negligible weight loss over 25 days. In comparison, the hybrid membranes containing silica experienced an initial weight loss of about 10–12% after 3 days. This is likely due to the release of silica from the membrane surface rather than the biodegradation of the membrane itself, as observed with PCL-CHT/MSN-Daidz samples, where the silica release also promoted the release of the preloaded compound. Indeed, the weight loss of the hybrid membrane PCL-CHT/MSN-Daidz over time correlates with the daidzein release (Figure 8a), which shows a peak within the first 48 h, followed by a slow and sustained release thereafter.

The swelling index of polymeric membranes is a key parameter in tissue engineering applications, as it facilitates nutrient transport and waste removal, which are essential for tissue repair and regeneration. As shown in Figure 8c, the swelling ratios of the hybrid membranes were evaluated at 37 °C over predetermined time intervals and compared with those of the PCL-CHT membranes. All membrane types exhibited time-dependent swelling, with values increasing until reaching a plateau at approximately 48 h. The hybrid membranes demonstrated a significantly higher degree of swelling, particularly within the first 48 h. After 48 h, the swelling ratios of the PCL-CHT, PCL-CHT/MSN, and PCL-CHT/MSN-Daidz membranes were 60 ± 5%, 68 ± 11%, and 67 ± 6%, respectively. This enhanced swelling capacity is attributed to the presence of MSNs, which increase the membranes hydrophilicity and porosity, thereby facilitating greater water uptake. Compared to other materials like PLGA (low swelling) and some forms of collagen (which may swell excessively, ∼700–2000%), leading to hydrogel instability or loss of structural integrity, the moderate swelling of the PCL-CHT/MSN membrane balances fluid uptake and mechanical stability. In contrast, alginate and silk fibroin materials exhibit very high swelling capacities (>700–1000%), making them ideal for highly exudative wounds but potentially less suitable for wounds requiring structural support. The moderate swelling of the PCL-CHT/MSN system, aided by the presence of chitosan and mesoporous silica, provides a good balance between exudate management and scaffold durability.

Maintaining an optimal moisture balance at the wound interface is critical for effective healing. To assess the barrier properties and breathability of the developed membranes, the water vapor transmission rate (WVTR) was measured over a 72 h period. An ideal wound dressing should exhibit a WVTR that supports a moist environment while preventing exudate accumulation or desiccation.

The WVTR profiles of the PCL-CHT, PCL-CHT/MSN, and PCL-CHT/MSN-Daidz membranes are listed in Figure 8d. All samples demonstrated an initial increase in WVTR over the first 8 h, followed by a slight inflection and eventual stabilization, indicating the establishment of a steady-state moisture flux. Membranes incorporating MSNs showed a marked increase in WVTR across all time points relative to the PCL-CHT control. This enhancement is attributed to the increased hydrophilicity and porosity conferred by the MSNs, which likely facilitated more efficient water vapor diffusion through the membrane matrix. Quantitatively, the inclusion of MSNs resulted in an increase in WVTR by approximately 12.5% to 48% after 7 h compared to the neat PCL-CHT membrane. However, after 48 h, the PCL-CHT, PCL-CHT/MSN, and PCL-CHT/MSN-Daidz membranes exhibited WVTR values of 11.3 ± 3.0, 32.2 ± 4.4, and 31.9 ± 6.1 g/ m^2^· h, respectively. These values fall within the WVTR range for wound dressings, which provide balanced moisture permeability and breathability while minimizing the risk of maceration or dehydration. Thus, the incorporation of MSNs into the PCL-CHT matrix significantly enhances the functional properties of the hybrid membranes by increasing membrane porosity and microchannel formation, facilitating vapor diffusion, and making them promising candidates for advanced wound management applications. However, the PCL-CHT/MSN membrane exhibited a moderate WVTR, lower than silk fibroin (∼87 g/m^2^·h) and alginate-based composites (up to 175  g/m^2^·h),^40,41^ but significantly higher than pure PCL, PLGA membranes, or some CHT polymers,^42^ which are typically hydrophobic with poor permeability. While it does not reach the upper range ideal for heavily exuding wounds, the WVTR of the PCL-CHT/MSN membrane is suitable for moderate-exudate wounds.

### Cell Responses to the Epidermal Membrane Systems

The in vitro cell responses were evaluated in epidermal membrane systems composed of human keratinocytes and the developed PCL-CHT membranes, as well as hybrid membranes with silica (PCL-CHT/MSN) and daidzein-preloaded silica (PCL-CHT/MSN-Daidz). Moreover, to simulate the continuous and prolonged release of daidzein from PCL-CHT/MSN-Daidz, human keratinocytes on silica hybrid membranes were treated with 50 μM of daidzein administered in the culture medium (PCL-CHT/MSN + Daidz). As reported in Figure 9a, human keratinocyte viability was sustained over 14 days in all the developed epidermal membrane systems, exhibiting significant cell proliferation after 7 days of culture. Cell viability increased up to 14 days, with the exception of the cells cultured on the hybrid membrane with silica and continuously treated with 50 μM of daidzein directly administered in the culture medium (PCL-CHT/MSN + Daidz).

**Figure 9:**
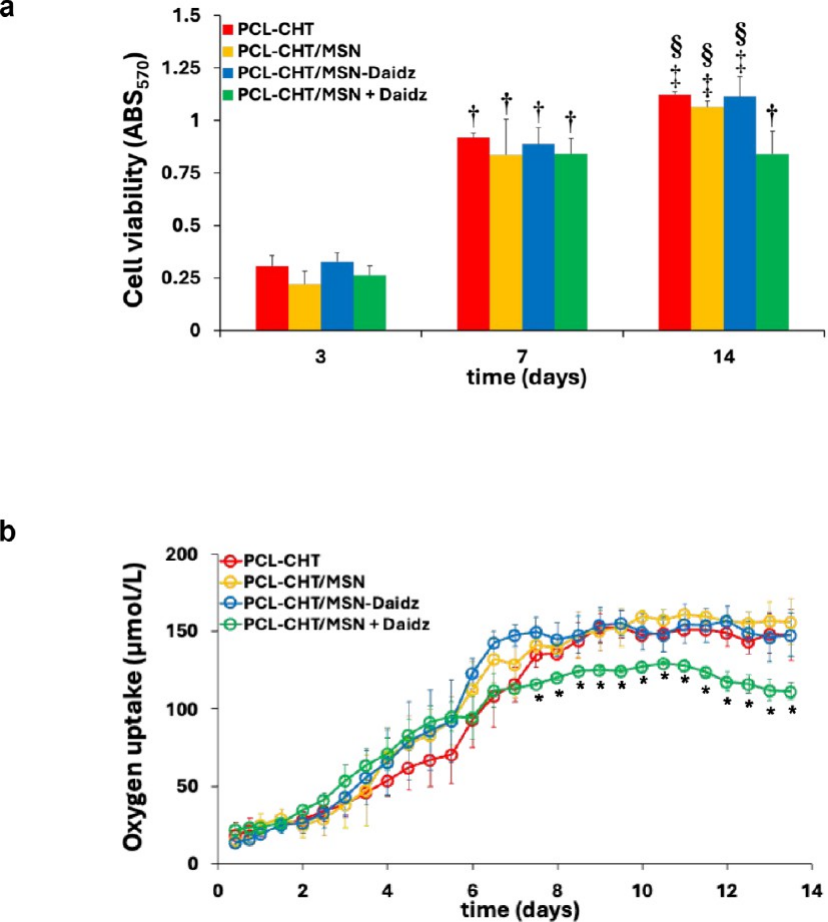
Viability (a) and oxygen uptake (b) of human keratinocytes cultured on native membranes without silica (PCL-CHT), hybrid membranes with silica (PCL-CHT/MSN), hybrid membranes with daidzein-preloaded silica (PCL-CHT/MSN-Daidz), and on PCL-CHT/MSN in the presence of daidzein 50 μM in the culture medium (PCL-CHT/MSN + Daidz). Data statistically significant according to ANOVA followed by the Bonferroni *t* test (*p* < 0.05): (†) vs day 3, (‡) vs day 3 and 7, on the same substratum; (§) vs PCL-CHT/MSN + Daidz, (*) vs all the substrates, at the same day of culture.

The oxygen uptake rate (OUR) by human keratinocytes in the developed epidermal membrane systems was continuously monitored for up to 14 days of culture. Oxygen is one of the main nutrients for living cells and is essential for carrying out metabolic activities. In vitro limitations to oxygen transport, due to its lower solubility in the aqueous phase compared to the gaseous phase, can cause a loss of cellular viability. In the developed epidermal models, oxygen consumption is representative of an active and functional metabolic state of cells in vitro. As reported in Figure 9b, oxygen consumption rises over time, leveling off at day 7 in all epidermal membrane systems. Notably, the highest oxygen consumption activity was observed in the hybrid membrane PCL-CHT/MSN, achieving values of 161 ± 3 μmol/L at day 11. Human keratinocytes cultured on the silica hybrid membranes and treated with 50 μM of daidzein in the medium (PCL-CHT/MSN + Daidz), after 7 days, exhibited a significantly lower oxygen uptake rate than that observed for the cells cultured on the other membranes. These results, consistent with the simultaneous decrease of cell viability (Figure 9a), highlight that human keratinocytes continuously treated with daidzein in the culture medium reduce their oxygen uptake over time, which is representative of a decreased energy demand and reduced metabolic functions. The hybrid membrane with daidzein-preloaded silica (PCL-CHT/MSN-Daidz) gradually releases daidzein, with a maximum peak of 88.9 μM/cm^2^_membr_ in the first 48 h (Figure 8a). In contrast to external treatment with 50 μM of daidzein in the culture medium, the controlled release from the preloaded membranes results in a higher release of daidzein within 48 h, without causing any impairment to cell viability during this period. To evaluate possible differences in the cell cycle, the percentage distribution of human keratinocytes in G1, S, and G2/M phases on various substrates and treatments was subsequently analyzed by cytofluorimetry after 7 days of culture. As shown in Figure 10, for the epidermal cells cultured on the native PCL-CHT membrane, the S phase of DNA synthesis and replication is extremely low compared to the cells cultured on the hybrid membranes containing silica. Both the treatments with daidzein preloaded in the hybrid membranes (PCL-CHT/MSN-Daidz) and administered in the culture medium (PCL-CHT/MSN + Daidz) evidenced a sustained increase in the synthesis phase S and a reduced preparatory phase G1. This shift in cell cycle distribution strongly suggests that daidzein promotes the G1-to-S phase transition and enhances keratinocyte proliferation. This finding is further supported by Western blot analysis showing upregulation of cyclin D1, a key positive regulator of the G1/S transition, in cells cultured on PCL-CHT/MSN-Daidz membranes (Figure 11a).

**Figure 10:**
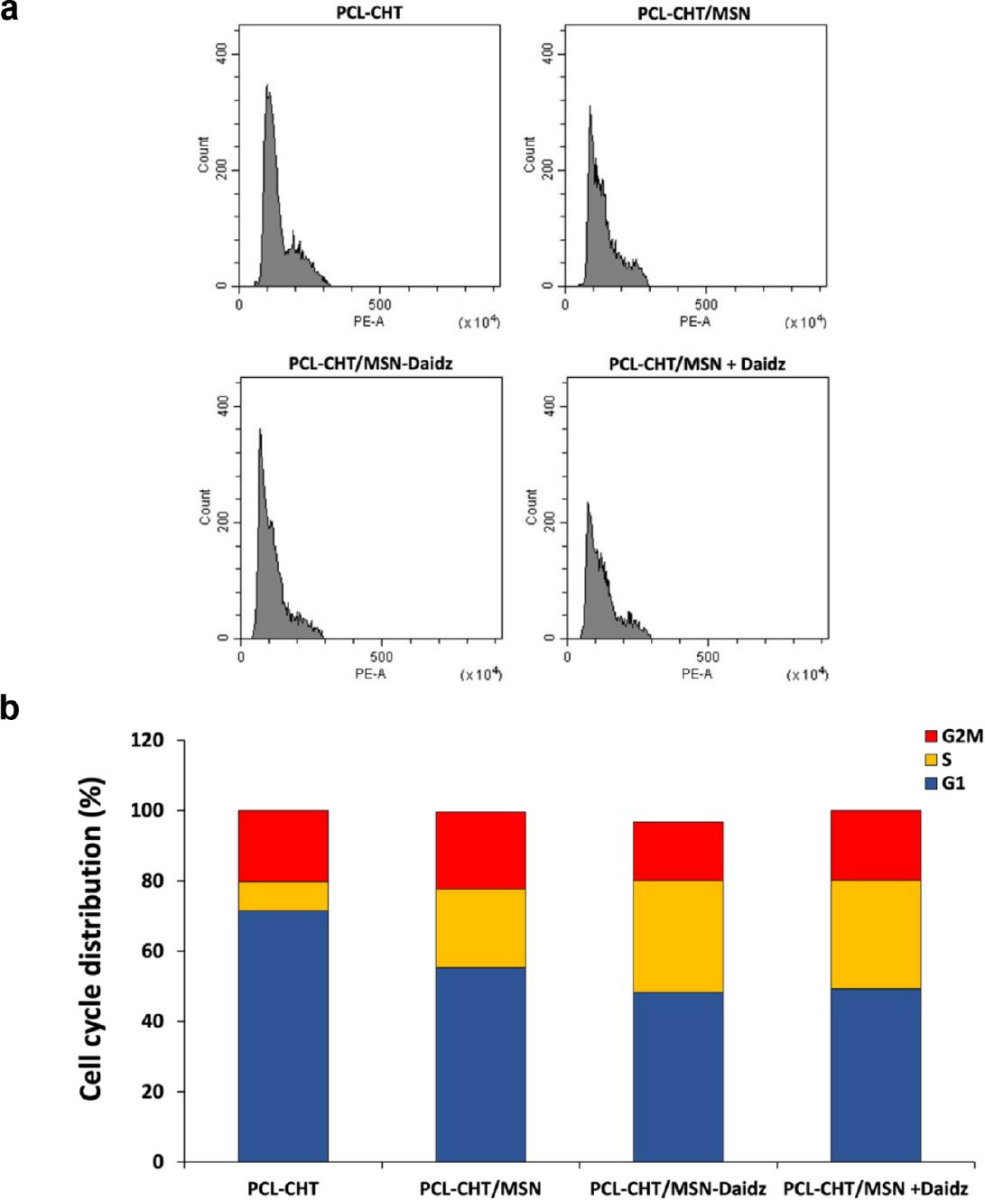
Cell cycle (a) and cell cycle distribution percentages (b) by FACS analysis of human keratinocytes after 7 days of culture on native membranes without silica (PCL-CHT), hybrid membranes with silica (PCL-CHT/MSN), hybrid membranes with daidzein-preloaded silica (PCL-CHT/MSN-Daidz), and on PCL-CHT/MSN in the presence of daidzein 50 μM in the culture medium (PCL-CHT/MSN + Daidz).

**Figure 11:**
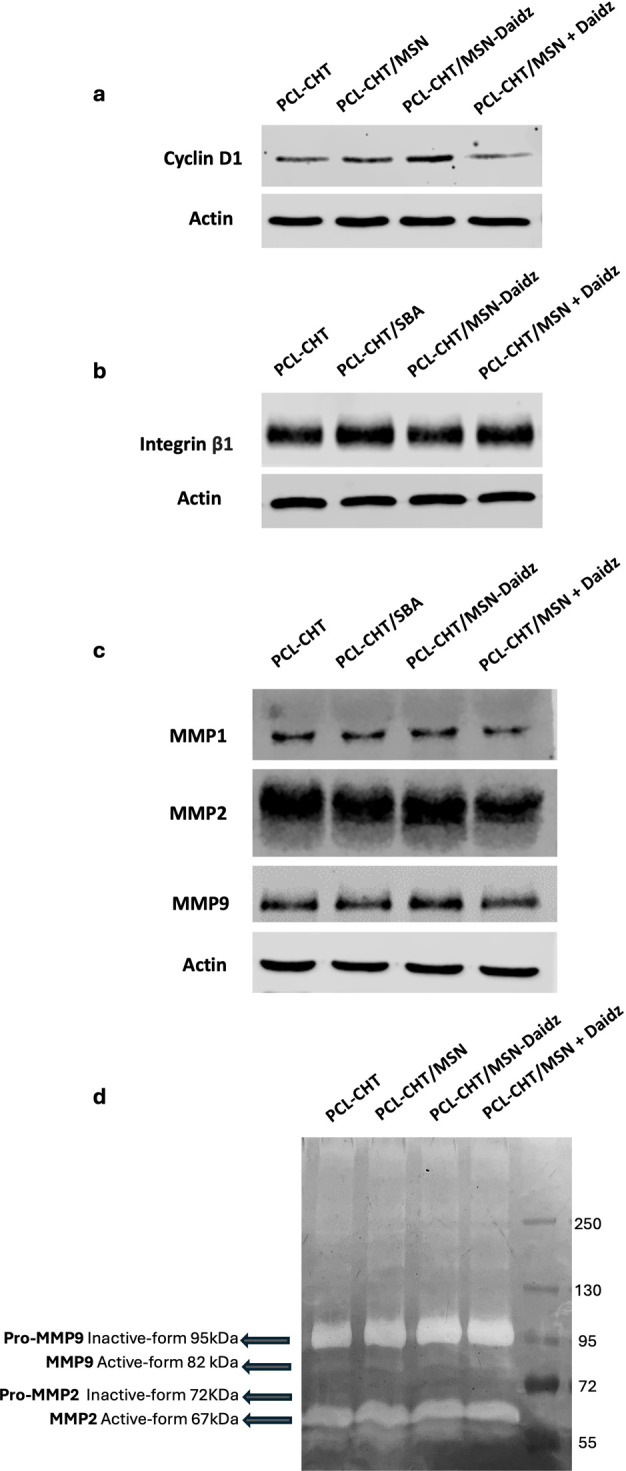
Western blotting of (a) cyclin D1, (b) integrin β1, and (c) MMP1, MMP2, and MMP9 expressed by human keratinocytes after 7 days of culture on native membranes without silica (PCL-CHT), hybrid membranes with silica (PCL-CHT/MSN), hybrid membranes with daidzein-preloaded silica (PCL-CHT/MSN-Daidz), and on PCL-CHT/MSN in the presence of daidzein 50 μM in the culture medium (PCL-CHT/MSN + Daidz). (d) Gelatin zymography of the gelatinolytic activity of both the proinactive and active forms of gelatinase MMP2 and MMP9 secreted by human keratinocytes after 7 days of culture on the same substrate and under the same culture conditions.

Indeed, Western blot analysis revealed a marked upregulation of cyclin D1 expression in human keratinocytes cultured for 7 days on hybrid PCL-CHT/MSN-Daidz membranes (Figure 11a). Together, these results indicate that daidzein release plays a primary role in driving proliferative responses, although synergistic effects with the hybrid membrane surface topography and polymer chemistry are also likely contributors. The significant expression of integrin β1 (Figure 11b), a receptor subunit that mediates cell–cell and cell–extracellular matrix adhesion, in all the epidermal membrane constructs highlights the keratinocyte adhesion on the developed membranes. Indeed, being recognized as a regulator factor in the initiation of keratinocyte terminal differentiation,^43^ a high expression of integrin β1 corroborates the evidence that all the developed membranes provide biochemical and physical cues to boost epidermal maturation.

Since the matrix metalloproteinases (MMPs) produced by keratinocytes aid in cutaneous wound repair by degrading the extracellular matrix and regulating cell migration, we investigated the expression of MMP1, MMP2, and MMP9, which have been shown to play a role in wound re-epithelialization by disrupting the tight junctions initially formed between keratinocytes and the dermal matrix. A similar expression of MMP1, MMP2, and MMP9 was found in protein extracts of keratinocytes grown on the native PCL-CHT membrane, as well as on hybrid membranes with daidzein-preloaded silica (PCL-CHT/MSN-Daidz) and with daidzein administered in the culture medium (PCL-CHT/MSN + Daidz) (Figure 11c). This finding is important considering the contribution of MMP1 to the reduction of both normal and hypertrophic scars^44^ and the role of both MMP2 and MMP9, which are crucial in promoting keratinocyte migration and remodeling granulation tissue during wound healing.^45^ However, MMP2 after 7 days of culture is highly expressed in keratinocytes on all investigated membranes with respect to the other MMPs, since MMP2 is required for cell proliferation and survival while it inhibits differentiation,^45^ as opposed to MMP9, which, instead, modulates the extracellular matrix to help keratinocytes spread, migrate, and differentiate. Specifically, the expression of MMP9 appears more pronounced in PCL-CHT/MSN-Daidz, indicating enhanced stratification and epidermal remodeling. These results were corroborated by a zymography assay conducted on the conditioned media harvested from keratinocytes grown in the different substrates and conditions. The secretion and the gelatinolytic activity of both the proinactive and active forms of gelatinase MMP2 and MMP9 are visible as clear areas of degradation over the dark gel (Figure 11d). The active form of MMP2 is highly marked with respect to the proinactive one, unlike MMP9 where the proinactive form is more pronounced than the active one.

Confocal laser scanning microscopy revealed successful differentiation of human keratinocytes after 7 days of culture on native PCL-CHT membranes. A well-defined, three-dimensional, multilayered epidermal structure was observed (Figure 12a,b). Cells in the basal layer adhered to the membrane surface, forming basal lamina cells. These basal keratinocytes exhibited a round morphology and expressed cytokeratin 18 (CK18), which is indicative of proliferative basal cells. Progressing toward the upper layers, keratinocytes became increasingly enlarged and flattened, consistent with differentiation. The outermost layers showed elevated expression of cytokeratin 1 (CK1), a marker characteristic of suprabasal epidermal cells, confirming the maturation of the stratum corneum.

**Figure 12:**
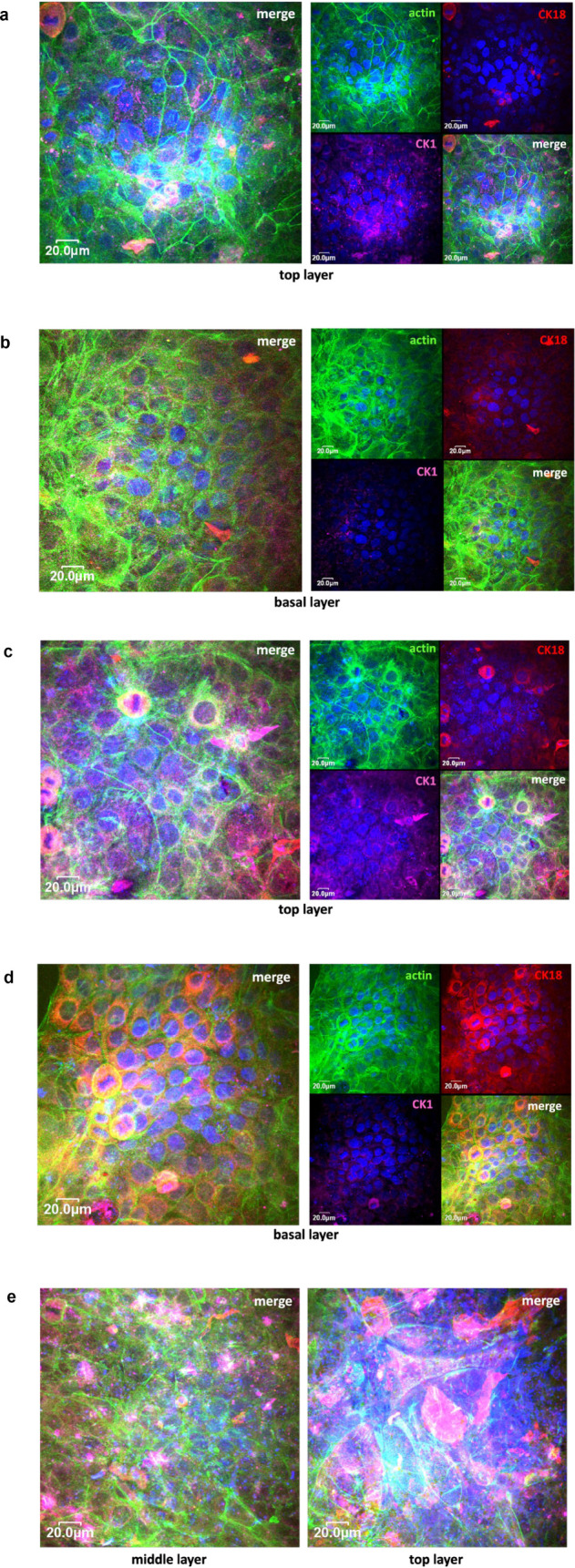
Cell morphology by CLSM images of human keratinocytes after 7 days of culture on native PCL-CHT membranes (a,b), hybrid PCL-CHT/MSN membranes (c,d), and hybrid PCL-CHT/MSN-Daidz membranes (e). Cells were visualized for actin (green), CK1 (magenta), CK18 (red), and nuclei (blue).

In contrast, keratinocyte stratification and complete epidermal differentiation, including the formation of a terminal stratum corneum, were more pronounced on the PCL-CHT/MSN membrane (Figure 12c,d). Basal keratinocytes exhibited sustained CK18 expression and retained a round morphology at the membrane interface, while flattened apical cells showed increased CK1 expression. These findings are attributable to the hybrid membrane unique meso- and nanoporous architecture resulting from silica nanoparticle incorporation. This topographical feature enhances keratinocyte adhesion, proliferation, and stratification. Furthermore, MSNs improve membrane hydrophilicity, modulating cell-material interactions and potentially activating signaling pathways involved in epidermal differentiation.

A comparable pattern of differentiation was observed for daidzein-loaded hybrid membranes (PCL-CHT/MSN-Daidz; Figure 12e). Keratinocytes in the intermediate spinous and granular layers displayed cuboidal morphology and coexpression of CK18 and CK1, reflecting a transitional differentiation stage. This coexpression marks the progressive migration of keratinocytes from the basal layer to the surface, characterized by a gradual decline in CK18 and an increase in CK1. The enhanced epidermal development on these membranes likely results from the synergistic combination of controlled daidzein release, optimized surface morphology, and the bioactive nature of the polymer matrix.

Collectively, these results demonstrate that the incorporation of mesoporous silica nanoparticles and bioactive compounds into PCL-CHT membranes significantly promotes keratinocyte differentiation and stratification, providing a promising strategy for the engineering of biomimetic skin substitutes.

## Conclusions

Hybrid membranes composed of polycaprolactone (PCL), chitosan (CHT) polymers, and calcined mesoporous silica nanoparticles—both in their bare form and preloaded with the active compound daidzein—have been designed and developed for the creation of epidermal constructs using human keratinocytes. The surface properties of the prepared membranes, both physical-chemical and morphological-structural, favor the adhesion and growth of human keratinocytes, demonstrating cytocompatibility. The hybrid membrane with daidzein-preloaded silica releases a maximum of 88.9 ± 0.9 μM/cm^2^_membr_ of daidzein after 48 h. The matrix metalloproteinases MMP1, MMP2, and MMP9, which aid in cutaneous wound repair by degrading the extracellular matrix and regulating cell migration, were produced by keratinocytes after 7 days, corroborating the evidence that the developed hybrid silica membranes provide biomimetic cues to facilitate epidermal maturation. In particular, the expression of MMP9 appears more pronounced in PCL-CHT/MSN-Daidz, indicating enhanced stratification and epidermal remodeling. Daidzein modulates cell cycle progression, promoting the G1-to-S phase transition and upregulating cyclin D1, potentially enhancing the coordination of tissue remodeling and repair by regulating the balance between proliferation and differentiation.

Morphological and functional evaluations of the developed constructs underscore the synergistic role of the structural and physical-chemical properties of the hybrid membranes. The incorporation of silica nanoparticles enhanced the surface texture and hydrophilicity, promoting cell adhesion and differentiation. These membranes successfully supported the formation of a complete, multilayered epidermis, as evidenced by the high expression of cytokeratins—CK18 in the basal lamina and CK1 in the superficial stratum corneum—reflecting proper epidermal maturation. These findings demonstrate the potential of hybrid membranes as effective scaffolds for skin tissue engineering applications.

## Supplementary Material


