# Supplementary material for: Mesoporous Silica-Loaded PCL-CHT Hybrid Membranes for Skin Regeneration
Source: ACS Appl Mater Interfaces. 2025 Aug 11;17(33):46651–66. doi: 10.1021/acsami.5c09164 (PMC12371686; doi:10.1021/acsami.5c09164)
Supplement: Supplementary file 1 [file am5c09164_si_001.pdf]

# Supporting Information

## MESOPOROUS SILICA-LOADED PCL-CHT HYBRID MEMBRANES FOR SKIN REGENERATION

Simona Salerno[1], Sabrina Morelli[1], Andrea Vardè[1], Marzia De Santo[2,3], Camilla Longobucco[2,3], Angelica Spadafora[3], Gianluca Dell'Olio[3], Francesca Giordano[2], Catia Morelli[2,3], Antonella Leggio[2,3], Luigi Pasqua[3,4], and Loredana De Bartolo[1]

[1]Institute on Membrane Technology, National Research Council of Italy, ITM-CNR, via P. Bucci, cubo 17/C, I-87036 Rende (CS), Italy.

[2]Department of Pharmacy, Health and Nutritional Sciences University of Calabria, via P. Bucci, 87036 Rende (CS), Italy

[3]NanoSiliCal Devices srl, University of Calabria, 87036 Rende (CS), Italy

[4]Department of Environmental Engineering, University of Calabria, via P. Bucci, 87036 Rende, (CS), Italy

***Corresponding authors**

Loredana De Bartolo, E-mail: l.debartolo@itm.cnr.it

Simona Salerno, E-mail: s.salerno@itm.cnr.it

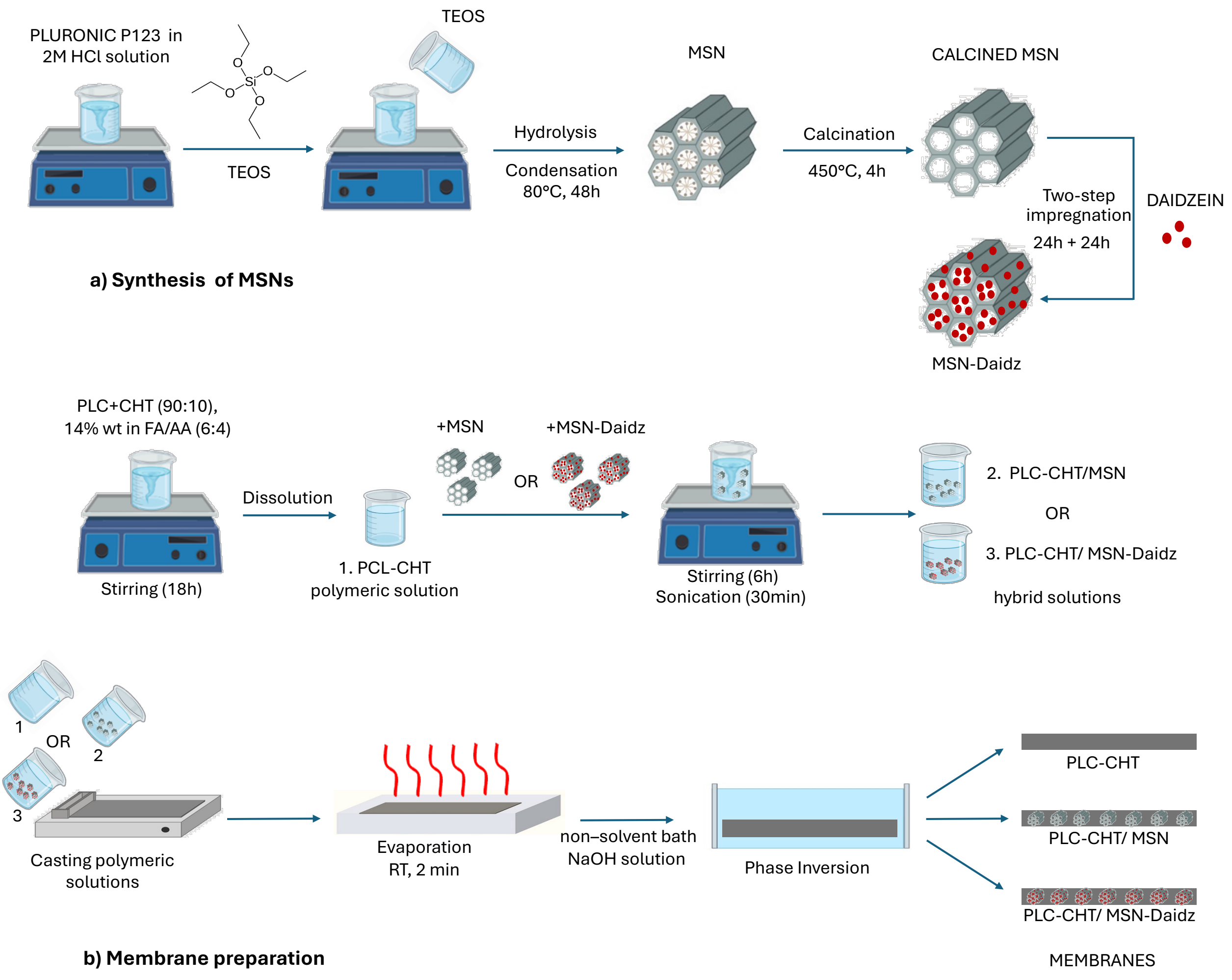


**Figure S1.** Schematic illustration of (a) MSNs synthesis and (b) membranes preparation.

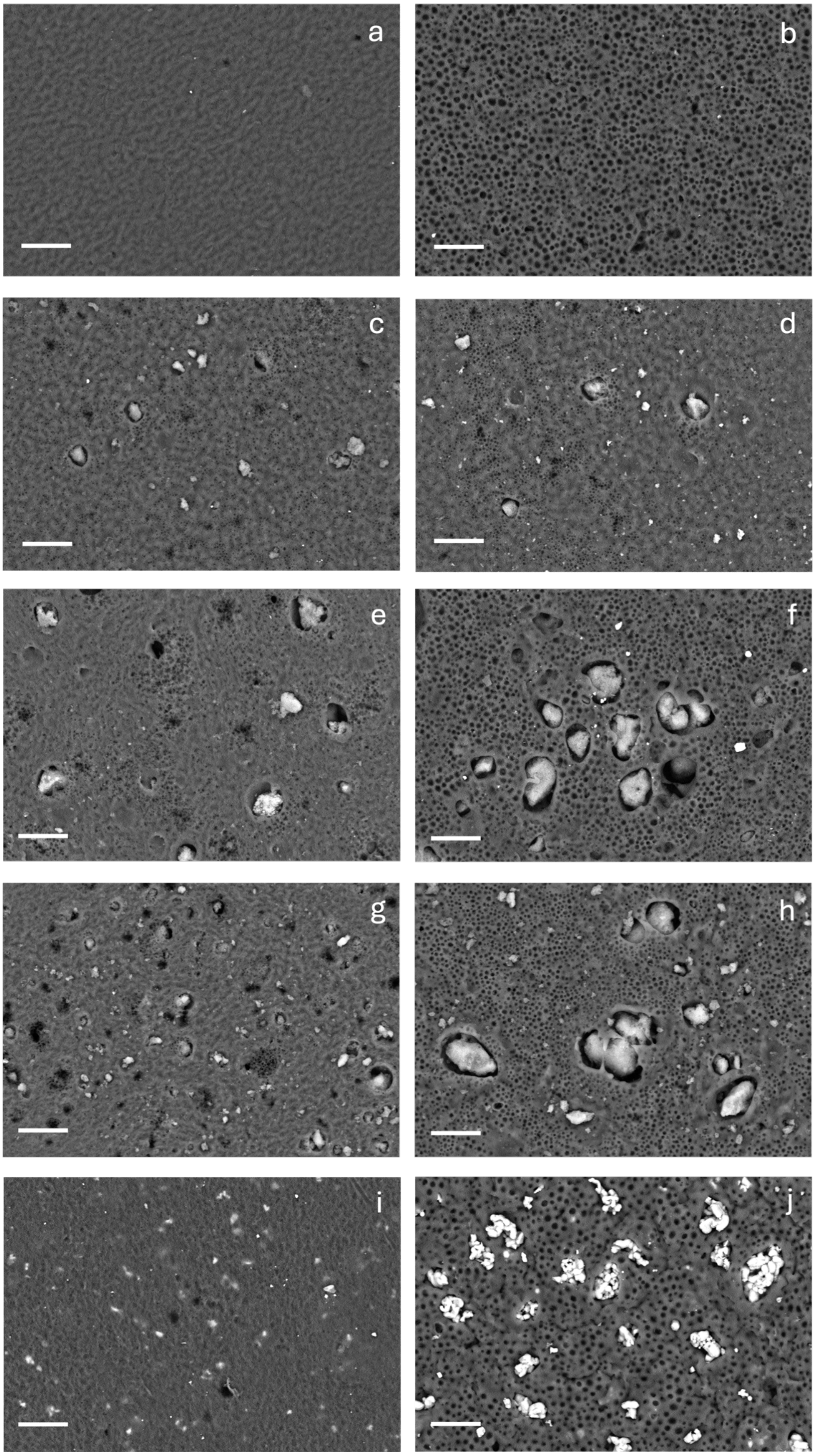


Figure S2. SEM micrographs in the BSD signal of top (a, c, e, g, i) and bottom surfaces (b, d, f, h, j) of the PCL-CHT (a-b) and hybrid PCL-CHT/MSN membranes with molar ratio of 20:1 (c-d), 10:1 (e-f), 7.5:1 (g-h), 5:1 (i-j). Scale bar: 25 μm.

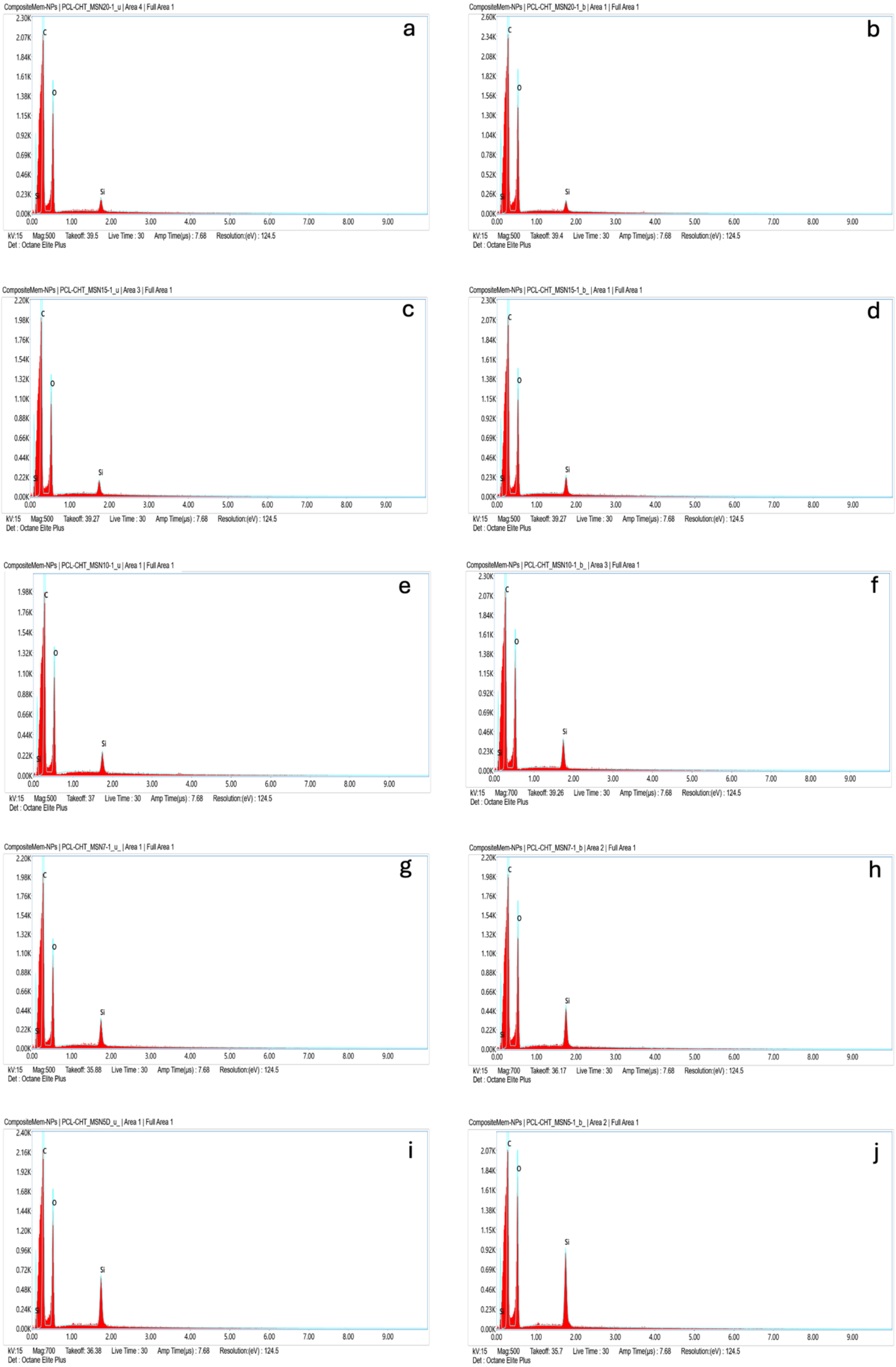


**Figure S3.** SEM-EDS spectra of top (a, c, e, g, i) and bottom surfaces (b, d, f, h, j) of the hybrid PCL-CHT/MSN membranes with molar ratio of 20:1 (a-b), 15:1 (c-d), 10:1 (e-f), 7.5:1 (g-h), 5:1 (i-j).

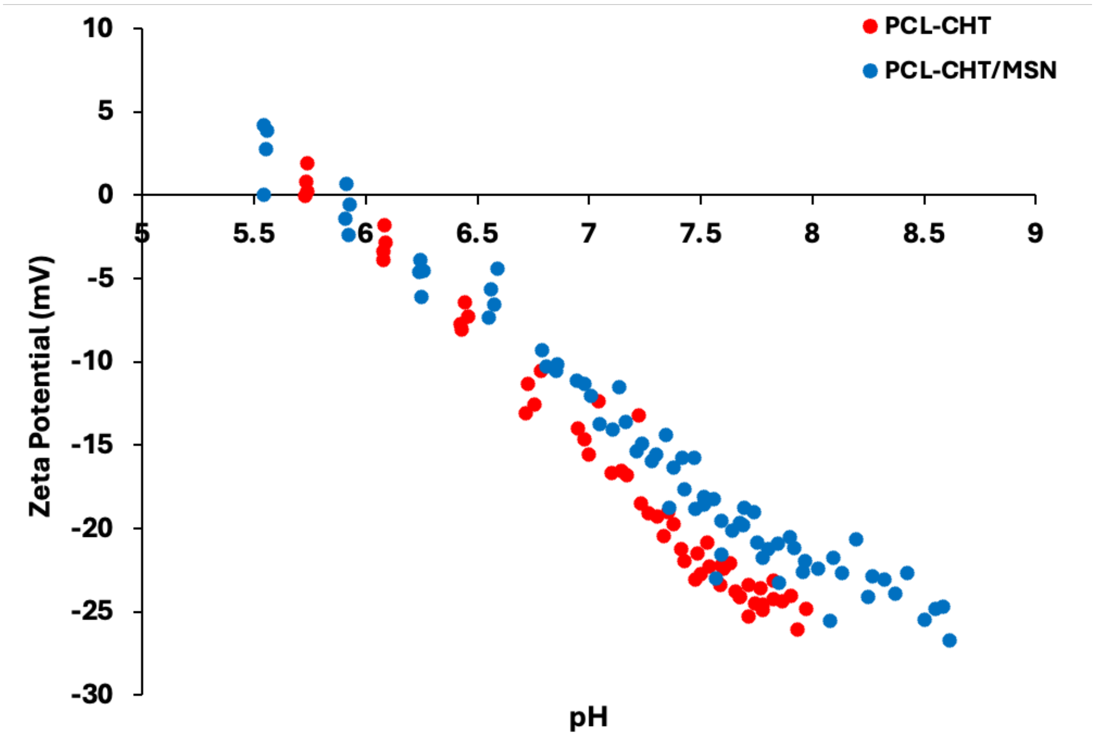


**Figure S4.** Zeta potential of PCL-CHT and PCL-CHT/MSN membranes at different pH.
